# Trends in Demographics Among Biosafety Professionals: A Decade-Long Analysis

**DOI:** 10.1089/apb.2024.0037

**Published:** 2025-08-29

**Authors:** An Tran

**Affiliations:** Environmental Science and Health, University of Nevada, Reno, Nevada, USA.

**Keywords:** biosafety professionals, demographic trends, salary trends, longitudinal analysis, sociodemographic factors, professional development

## Abstract

**Introduction:**

This study examines demographic trends among biosafety professionals from 2013 to 2024, focusing on changes in age, race, education, experience, and income. The goal is to inform educational and targeted interventions for the evolving needs of the biosafety profession.

**Methods:**

Surveys were conducted in 2013, 2016, 2020, 2023, and 2024 among ABSA International affiliates and Institutional Biosafety Committee contacts. Longitudinal analyses using Chi square, Cochran–Armitage tests, logistic regression, and one-way analysis of variance were performed to assess demographic shifts. The significance level was set at 0.05.

**Results:**

The study revealed demographic and professional shifts within the biosafety field from 2013 to 2024. There was a marked increase in the proportion of older, more experienced professionals and those holding doctoral degrees. However, female representation declined, and the field remained predominantly White, with minimal change in racial and ethnic diversity. Although income increased significantly, the rise was less substantial when adjusted for inflation. Education level and experience consistently predicted higher income, while the influence of age and gender on salary varied over time.

**Discussion:**

The aging workforce and higher educational attainment highlight the need for policies to attract younger and more diverse talent in the biosafety profession. Income growth, though nominally significant, showed modest real gains after inflation adjustment. Understanding these trends is crucial for sustaining and developing the biosafety profession.

## Introduction

*Biosafety* is defined as “the application of practices, controls, and containment infrastructure that reduces the risk of unintentional exposure to, contamination with, release of, or harm from pathogens, toxins, and other associated biological materials.”^1^ However, some experts argue that this definition is too narrow and fails to capture the full scope of responsibilities involved.^2^

Biosafety officers are responsible for ensuring the safe handling and management of biological materials at various types of institutions.^3,4^ They help prevent exposure to infectious agents and biohazards by overseeing compliance with safety regulations, conducting risk assessments, and implementing biosafety programs. Their work involves collaborating with researchers, developing safety protocols, responding to biological incidents, interfacing with upper management, ensuring adherence to regulations, conducting laboratory surveys, managing biological waste management, ensuring secure facilities, and other important duties. Biosafety officers play an important role in protecting both personnel and the environment from biological hazards, making their work vital to public health and safety.^5^

Over the past 20 years, numerous regulations and policies have significantly impacted the roles and responsibilities of biosafety professionals. Notable examples include:

- The 2002 Select Agent Regulations, introduced under the *Public Health Security and Bioterrorism Preparedness and Response Act*, significantly expanded the duties of biosafety officers.^6^ With these new regulations, biosafety professionals were responsible for ensuring strict compliance with new requirements governing the possession, use, and transfer of select agents and toxins. This mandate required the implementation of rigorous security measures, maintaining accurate inventories and conducting background checks. Furthermore, biosafety officers were often tasked with developing, implementing, and managing occupational health programs including health monitoring, vaccination, medical surveillance, training and education to protect the health and well-being of personnel working with these agents, ensuring that the necessary protective measures were established and followed. In addition, they were charged with managing mandatory training for personnel handling regulated agents.
- The 2014 *U.S. Government Policy for Institutional Oversight of Life Sciences Dual-Use Research of Concern (DURC)* further increased the responsibilities of biosafety officers.^7^ They were tasked with identifying and monitoring DURC and ensuring strict adherence to guidelines designed to mitigate misuse of biological materials. This involved developing and implementing risk mitigation plans, providing researcher training in DURC policies, and facilitating communication between institutional review entities (IREs) and Federal agencies.
- The 2017 *Recommended Policy Guidance for Potential Pandemic Pathogen Care and Oversight* introduced additional requirements.^8^ Biosafety officers were expected to conduct more stringent risk assessments and provide enhanced oversight for research involving potential pandemic pathogens. This included ensuring that research proposals underwent rigorous review processes to assess risks and benefits, implementing appropriate biosafety measures, and engaging in ongoing monitoring and compliance efforts to prevent accidental release or misuse of high-risk pathogens.
- The 2024 *U.S. Government Policy for Oversight of Dual-Use Research of Concern and Pathogens with Enhanced Pandemic Potential* (PEPP) introduced new biosafety oversight requirements.^1^ Biosafety officers are required to implement new policies and procedures to assess and mitigate risks associated with DURC and PEPP research. This includes expanded coordination with Federal agencies, increased scrutiny of research proposals involving biological agents, and the implementation of advanced safety and security protocols. In addition, biosafety professionals are responsible for maintaining detailed documentation and ensuring continuous compliance with evolving biosafety, biosecurity, and risk management requirements.

These regulatory and policy changes have shifted the focus of biosafety professionals from purely safety-oriented practices to a greater emphasis on security. This transition has required the adoption of new methodologies for regulatory compliance, including more comprehensive risk assessments and ongoing professional development. The COVID-19 pandemic further broadened the responsibilities of biosafety professionals by highlighting their critical role in emergency response and public health protection.^9–13^ As their duties continue to evolve, biosafety professionals must stay abreast of emerging threats and continually adapt their practices to meet heightened security demands. Over the past decade, the field of biosafety has undergone significant transformations driven by technological advancements, evolving regulatory landscapes, shifting workforce responsibility, and the COVID-19 pandemic.

Understanding that the current state of diversity and inclusivity in the biosafety profession can inform policies, education initiatives, and recruitment strategies aimed at fostering a more innovative workforce. Analysis of workforce demographics and trends enables more targeted and effective efforts to address existing challenges and supports the development of innovative solutions in this evolving field.

Previous research by Gillum, Fletcher, and co-authors (2013, 2014, 2016, and 2020) has consistently indicated that factors such as educational attainment, years of professional experience, specific biosafety responsibilities, reporting structures, and gender significantly influence salary within the field.^14–17^ Understanding these determinants over time is critical for guiding workforce development strategies and policymaking. Such policies may include directives that impact the recruitment, training, and certification of biosafety professionals and initiatives to support diversity, equity, and inclusion. Funding for educational programs and setting regulatory standards can also help ensure consistent safety practices across institutions. This analysis seeks to provide detailed insights that will enable institutions to respond to emerging trends, enhance their ability to attract and retain top talent, and cultivate a supportive and equitable work environment. In addition, it aims to empower biosafety professionals with the knowledge necessary for effective career advancement, ensuring they are well equipped to navigate the evolving demands of their field.

## Method

This research explores the sociodemographic evolution of biosafety professionals from 2013 to 2024, examining factors such as education, experience, age, income, race, and gender, alongside changes in professional responsibilities. The findings aim to guide stakeholders in enhancing workforce support, development, and diversity, as well as in shaping policies that reflect these evolving trends.

### Data Source

Original data were provided by the authors of the previously mentioned research.^14–17^ These data were collected through surveys administered in 2013, 2016, 2020, 2023, and 2024 to individuals affiliated with ABSA International and those listed as Institutional Biosafety Committee contacts, in accordance with the National Institutes of Health’s (NIH) funding policy titled *NIH Guidelines for Research Involving Recombinant and Synthetic Nucleic Acid Molecules*.^18^ Despite the uneven intervals between data points, incorporating all available data provides the most comprehensive understanding of trends and changes over time while increasing the validity of the analysis. Participation in the original surveys was voluntary, and all Institutional Review Board (IRB) policies regarding human subject research were obtained and strictly adhered to. The data used in this study were deidentified, and the research was determined not to require human research protection oversight by the IRB at the University of Nevada, Reno (IRB no. 2236719).

Respondents were classified as biosafety professionals in the United States if they answered “Yes” to the questions, “Are you currently employed in a position with responsibility for biosafety?” and “Are you currently working in the United States?” The survey also included questions related to demographics (e.g., gender, age, race, ethnicity), socioeconomic status (e.g., highest degree or level of education, years of experience, income), and workplace characteristics (e.g., institution category, percentage of biosafety responsibility, number of inspections per year, size of the biosafety team, number of employees reporting to them, percentage of time spent on data entry, record-keeping tasks).

To ensure compatibility across the years, age data from 2016 were excluded as they were categorized differently (e.g., 25–34, 35–44, 45–54, 55–65, and >65 years). Age data from 2013 and 2020 were recategorized to match the 2023 format (i.e., 21–30, 31–40, 41–50, 51–60, 61–70, and >70 years). The 2013 survey did not include questions about race, and the 2013 and 2016 surveys did not include questions about ethnicity. The 2023 survey did not include questions about the biosafety team or the number of employees reporting to respondents. In addition, questions about the number of inspections conducted were not included in the 2020, 2023, and 2024 surveys.

The percentage of biosafety responsibility was not included in the 2024 survey. However, data on gender, years of experience, level of education, income, and institution categories were consistently available across all five survey years (i.e., 2013, 2016, 2020, 2023, and 2024). For the level of education, respondents who indicated “associate degree,” “some college but no degree,” and “training/vocational training” were classified as having a “high school degree or equivalent.” Income data were available in actual numbers for 4 years (2013, 2016, 2020, and 2024), and these were analyzed in this study to examine the variables influencing income changes over time.

### Statistical Analysis

Respondents were classified as having responsibility for biosafety activities and were working in the United States. The total sample comprised 2437 responses, distributed as follows: 356 in 2013, 580 in 2016, 572 in 2020, 409 in 2023, and 520 in 2024. Since respondents were not required to answer every question and had the option to skip questions or select “prefer not to say,” the total number of responses varied for each question. Responses of “prefer not to say” were excluded from the descriptive analysis.

Descriptive statistics were computed for 12 variables for each year. To test for differences in these variables, Chi square tests were conducted. Based on the results, dichotomous variables were created at significant thresholds for further analysis: education level (doctorate vs. not doctorate, including high school diploma, bachelor’s degree, and master’s degree), income range (<$100,000 vs. >$100,000), experience (<15 years vs. >15 years), age (20–39 vs. >39), race (White vs. non-White), institution category (academic vs. other), percentage of time spent on biosafety responsibilities (1–50% vs. 51–100%), number of professionals in the biosafety team (1–3 vs. >3), number of employees reporting to respondents (none to 1 vs. >1), number of inspections conducted annually (none to 100 vs. >100), and percentage of time spent on data entry and record-keeping tasks (1–50% vs. 51–100%).

Chi square tests were used to compare the differences in these dichotomous outcomes, and the Cochran–Armitage test was employed to assess linear trends in the proportions of these variables over the years. To further investigate changes from specific years to reference years (2013 and 2016), logistic regression models were conducted.

ANOVA tests were performed to analyze the differences in actual income data (without inflation adjusted) and inflation-adjusted income data across 4 years. The inflation-adjusted income was calculated using the consumer price index^19^ of the base year (2024) compared with other years, allowing for a comparison over time without the effects of inflation. In addition, linear regression analyses were conducted for each year to examine the influence of sociodemographic factors on income trends. A further linear regression was performed with the year as an independent variable to assess its contribution as a predictor of income for biosafety professionals, along with other predictors, including gender, age, and level of education.

## Results

### Demographics

Analysis of demographic changes between 2013 and 2024 for biosafety professionals shows several significant differences (Table 1).

**Table 1.** Differences of biosafety professionals by demographics over years

	2013 (%)	2016 (%)	2020 (%)	2023 (%)	2024 (%)	Chi square
Gender						
Female	56.00	55.96	54.60	51.59	47.95	5.81
Male	44.00	44.04	45.40	48.41	52.05	
Age						
20–29	4.50		3.37	1.27	1.21	52.36***
30–39	22.52		23.98	14.01	16.19	
40–49	26.73		31.62	32.80	33.6	
50–59	35.14		25.75	30.25	27.53	
60–69	10.21		13.68	16.88	17.41	
70 or more	0.90		1.60	4.78	4.05	
Race						
White		87.62	86.67	90.3	87.87	21.22*
Black or African		2.63	3.53	3.34	2.93	
Asian or Native Hawaiian or Pacific Islander		9.01	7.45	5.02	6.28	
American Indian		0.38	0.59	1.34	0.42	
Other		0.38	1.76	0.00	2.51	
Ethnicity						
Hispanic or Latino or Spanish Origin			7.32	9.09	7.63	11.48*
Not Hispanic or Latino or Spanish Origin			89.02	90.91	89.83	
Other			3.66	0.00	2.54	

*Notes*; *p < 0.05, **p < 0.01, ***p < 0.001.

The distribution of gender among biosafety professionals remained relatively stable over the years (*χ^2^*([4] = 5.81, *p* = 0.21). There was a slight increase in the proportion of male professionals from 44% in 2013 to 52.05% in 2024. Conversely, the percentage of female professionals decreased slightly from 56% in 2013 to 47.95% in 2024, representing a shift toward a more even gender distribution in the field.

The age distribution shows significant changes over the years (*χ^2^*[15] = 52.36, *p* < 0.0001). There is a noticeable decrease in the proportion of younger professionals (20–39 years), dropping from 27.02% in 2013 to 17.4% in 2023. Conversely, there is an increase in older age groups, particularly among those aged 60 or older, rising from 11.11% in 2013 to 21.46% in 2024. The most represented age group in 2013 is the 50–59 age group at 35.14% which decreased over time to 27.53% in 2023.

The racial composition is significantly different over the years (*χ^2^*[12] = 21.23, *p* < 0.05). More than 85% of biosafety professionals in the United States were White with their proportion increasing from 87.62% in 2016 to 90.3% in 2023. The representation of Asian or Native Hawaiian or Pacific Islander professionals decreased from 9.01% in 2016 to 6.28% in 2024. Other racial groups, such as Black or African and American Indian, showed slight increases but remained a small proportion of the total.

There is a statistically significant change in the distribution of ethnicity(*χ^2^*[4] = 11.48, *p* < 0.05). The percentage of Hispanic or Latino or Spanish Origin professionals increased from 7.32% in 2020 to 9.09% in 2023 before decreasing to 7.63 in 2024. The proportion of non-Hispanic or Latino professionals remained high, with a slight increase from 89.02% in 2016 to 89.83% in 2024.

### Socioeconomic

Analysis of socioeconomic factors between 2013 and 2024 for biosafety professionals shows several substantial differences (Table 2).

**Table 2.** Differences of biosafety professionals by socioeconomic factors over years

	2013 (%)	2016 (%)	2020 (%)	2023 (%)	2024 (%)	Chi square
Experience						
<1 year	2.04	2.08	3.16	0.30	1.24	67.51^***^
At least 1–5	16.03	21.51	11.93	9.34	13.22	
At least 5–10	26.82	25.47	23.16	18.98	19.01	
At least 10–15	22.16	18.49	23.16	21.39	23.55	
>15 years	32.94	32.45	38.60	50.00	42.98	
Education						
High school degree or equivalent	1.46	1.55	3.15	0.91	0.41	78.08^***^
Bachelor’s Degree or equivalent	23.32	23.66	22.94	17.38	16.33	
Master’s degree or equivalent	48.40	44.39	37.13	32.32	33.06	
Doctorate	26.82	30.4	36.78	49.39	50.2	
Income						
<$20,000 USD	0.33	0.55	0.19	0.00	0.00	222.02^***^
At least $20,000 USD but <$40,000	2.62	0.55	1.88	0.34	0.00	
At least $40,000 USD but <$60,000	14.75	15.36	8.65	3.74	2.78	
At least $60,000 USD but <$80,000	23.28	28.5	22.74	12.93	13.43	
At least $80,000 USD but <$100,000	26.56	21.02	21.43	21.77	22.69	
At least $100,000 USD but <$120,000	16.72	16.64	14.66	19.39	4.63	
At least $120,000 USD but <$140,000	7.54	6.95	11.28	14.29	14.35	
At least $140,000 USD but <$160,000	6.23	5.12	8.65	9.86	9.72	
At least $160,000 USD but <$180,000	0.66	1.83	3.95	6.12	6.94	
At least $180,000 USD but <$200,000	0.66	0.91	2.26	4.76	4.63	
$200,000 USD or more	0.66	2.56	4.32	6.8	12.96	

^***^ *p* < 0.001.

The distribution of professional experience has shifted significantly (*χ^2^*[16] = 67.51, *p* < 0.0001). The proportion of professionals with <15 years of experience decreased from 67.05% in 2013 to 57.02% in 2023, in which professionals from 10 to 15 years of experience did not change much. Conversely, professionals with >15 years of experience increased markedly from 32.94% in 2013% to 50% in 2023.

There are significant changes in the educational level of biosafety professionals (*χ^2^*[12] = 78.08, *p* < 0.0001). The proportion of those with a high school degree or equivalent remained low but varied slightly, from 3.15% in 2020 before dropping to 0.41% in 2024. The percentage of professionals with either a bachelor’s degree or master’s degree decreased from 71.72% in 2013 to 49.4% in 2024. However, the proportion of professionals with a doctorate increased from 26.82% in 2013 to 50.2% in 2023.

Actual income distribution has also undergone significant changes (*χ^2^*[40] = 222.02, *p* < 0.0001). The percentage of professionals earning <$20,000 USD decreased to 0% in 2024 from 0.33% in 2013. Those earning $20,000 to $80,000 USD reduced from 67.21% in 2013 to 38.9% in 2024. However, those earning $80,000 to $100,000 USD remained relatively stable, slightly fluctuating around 21%. There was a significant increase in professionals earning >$100,000. Especially, higher income brackets, including those earning >$160,000 USD, saw increases, with just 1.98% in 2013 increasing to 24.53% in 2024.

Analysis of the actual income data collected from the survey revealed a significant difference in reported income in 4 years (*F*[3] = 30.44, *p* < 0.0001). The Tukey post hoc test indicated that the income of biosafety professionals in 2024 (*M* = $127,350, *SD* = $56,802) was significantly higher than the income reported in 2013 (*M* = $88,008.25, *SD* = $32,299.88), 2016 (*M* = $92,977, *SD* = $60,436), and 2020 (*M* = $103,721, *SD* = $47,299). In addition, the Tukey post hoc test showed that income in 2020 was significantly higher than in both 2013 and 2016. In contrast, analysis of the inflation-adjusted income revealed no significant differences over the 4 years (*F*[3] = 1.37, *p* = 1.37) (Table 3).

**Table 3.** Differences of income of biosafety professionals over the years

Year	Mean	SD	df	*F*-value	ηp^2^
Income					
2013	$88,008.25	$32,299.88	1600	30.44^***^	0.0541
2016	$92,977.32	$60,436.30			
2020	$103,721.61	$47,299.35			
2024	$127,350.93	$56,802.60			
Adjusted income					
2013	$117,924.28	$43,279.34	1600	1.37	0.0026
2016	$120,922.94	$78,601.32			
2020	$125,096.63	$57,046.38			
2024	$127,350.93	$56,802.60			

*Note:* ***p < 0.0001.

### Workplace and Position Characteristics

The institutional and position characteristics of biosafety professionals show several substantial differences (Table 4).

**Table 4.** Differences of biosafety professionals by institution and position characteristics over the years

	2013 (%)	2016 (%)	2020 (%)	2023 (%)	2024 (%)	Chi square
Institution category						
Academic (private)	17.61	17.46	16.94	16.46	15.89	17.03
Academic (public)	37.78	40.92	37.12	40.65	40.89	
Government	10.51	13.05	16.22	17.21	16.82	
Other	34.09	28.57	29.73	25.69	26.4	
Biosafety time						
1–25%	24.5	22.6	30.3	39.36	39.36	40.22^***^
26–50%	19.66	17.51	15.41	15.4	15.4	
51–75%	13.96	12.99	14.54	11	11	
76–100%	41.88	46.89	39.75	34.23	34.23	
Biosafety team						
1	29.86	27.43	21.83	0	18.23	36.19^**^
2	22.32	21.33	21.13	0	21.72	
3	15.65	13.9	11.97	0	12.06	
4	11.3	10.86	13.2	0	11.26	
5	20.87	26.47	31.87	0	36.73	
Employee reporting						
0	52.45	53.38	51.85			11.27
1	12.68	14.66	9.35			
2	7.49	7.89	9.52			
3	6.34	5.64	6			
4	21.04	18.42	23.38			
Inspection number						
<25	28.07	31.15				7.19
25–100	26.9	23.85				
101 or more	38.89	42.12				
We do not perform	6.14	2.88				
Data entry and record keeping responsibility						
None	2.7	0	5.58			63.26^***^
1–25%	56.76	54.77	54.84			
25–50%	26.73	32.86	25.74			
51–75%	10.51	10.07	10.16			
76–100%	3.3	2.3	3.68			

^**^ *p* < 0.001.

^***^ *p* < 0.0001.

The distribution of biosafety professionals across different institution categories has not shown significant changes over the years (*χ^2^*[12] = 17.03, *p* = 0.15). Academic institutions, both private and public, continue to employ a large proportion of biosafety professionals.

There is a significant change in the proportion of time biosafety professionals dedicate to biosafety duties over time (*χ^2^*[9] = 40.22, *p* < 0.0001). The proportion of professionals spending 1–25% of their time on biosafety tasks increased from 24.5% in 2013 to 39.36% in 2023. Conversely, those dedicating 76–100% of their time to biosafety tasks have decreased from 41.88% in 2013 to 34.23% in 2023.

The size of biosafety teams has also changed significantly (*χ^2^*[12] = 36.19, *p* < 0.001). The proportion of single-person teams decreased from 29.86% in 2013 to 18.23% in 2024. However, there is a notable increase in the proportion of five-person teams, rising from 20.87% in 2013 to 36.73% in 2024.

### Trend Analysis >11 Years

In the next phase of the analysis, we build on the initial Chi square tests by exploring trends across key characteristics. To assess whether there are significant trends at critical thresholds over time, we employ the Cochran–Armitage trend test for each characteristic (Table 5). In addition, logistic regression models are used to further investigate the relationship between these variables and year, with comparisons made to the reference years 2013 and 2016 (Table 6). This combined approach offers a more comprehensive understanding of the temporal patterns in the data.

**Table 5.** Trend analysis of sociodemographic factors over years

	Chi square	Cochran–Armitage test
Gender		
Female	5.81	−2.07^*^
Male		
Age		
20–39	23.99^***^	−3.48^**^
>39		
Race		
White	0.38	0.68
Other		
Ethnicity		
Hispanic and Latino	0.58	0.43
Other		
Experience		
<15 years	33.27^***^	−5.05^***^
>15 years		
Education		
Doctorate	39.40^***^	5.77^***^
Not doctorate		
Income		
<$100,000	98.4	−9.47
>$100,000		
Institution category		
Academic	2.39	−0.06
Other		
Time on biosafety		
<50%	20.55^**^	3.57^**^
>50%		
Biosafety team		
<3	25.46^***^	−4.94^***^
>3		
Employee reporting		
<1	5.67	−1.65
>1		
Inspection number		
<100	0.89	−0.94
>100		
Data entry and record keeping time		
<50%	0.64	−0.21
>50%		

^*^ *p* < 0.05.

^**^ *p* < 0.001.

^***^ *p* < 0.0001.

**Table 6.** Logistics regression of socioeconomic factors and job characteristics over years

Year	Year reference	OR	CI (low)	CI (high)
Gender (male vs. female)				
2016	2013	1.0	0.76	1.13
2020	2013	1.06	0.80	1.40
2023	2013	1.19	0.88	1.63
2024	2013	1.38	0.99	1.93
2024	2016	1.38^*^	1.02	1.86
Age (>39 vs. 20–39)				
2020	2013	0.98	0.73	1.33
2023	2013	2.05^**^	1.39	3.04
2024	2013	1.76^**^	1.17	2.64
2023	2020	2.09^***^	1.46	2.99
2024	2020	1.79^**^	1.23	2.61
Race (White vs. Other)				
2020	2016	0.92	0.64	1.32
2023	2016	1.32	0.83	2.09
2024	2016	1.02	0.64	1.63
Experience (>15 years vs. <15 years)				
2016	2013	0.98	0.73	1.31
2020	2013	1.28	0.97	1.70
2023	2013	2.04^***^	1.49	2.78
2024	2013	1.53^*^	1.09	2.16
2020	2016	1.31^*^	1.02	1.68
2023	2016	2.08^***^	1.57	2.76
2024	2016	1.57^**^	1.15	2.15
Education level (doctorate vs. not doctorate)				
2016	2013	1.11	0.77	1.61
2020	2013	1.30	0.91	1.87
2023	2013	2.5^***^	1.64	3.79
2024	2013	2.77^***^	1.75	4.39
Income range (>$100 K vs. <$100 K)				
2016	2013	1.07	0.80	1.44
2020	2013	1.68^**^	1.25	2.25
2023	2013	3.25^***^	2.33	4.55
2024	2013	3.24^***^	2.25	4.65
Institution category (academic vs. other)				
2016	2013	1.13	0.86	1.48
2020	2013	0.95	0.72	1.24
2023	2013	1.07	0.80	1.43
2024	2013	1.06	0.80	1.41
Biosafety time (<50% vs. >50%)				
2016	2013	0.88	0.65	1.11
2020	2013	1.07	0.82	1.39
2023	2013	1.53^*^	1.15	2.04
2023	2016	1.81^***^	1.39	2.35
Biosafety team (>3 vs. <3)				
2016	2013	1.26	0.94	1.67
2020	2013	1.73^**^	1.31	2.29
2024	2013	1.95^***^	1.44	2.64
2020	2016	1.38^*^	1.08	1.75
2024	2016	1.55^*^	1.18	2.03
Employee reporting (>1 vs. <1)				
2016	2013	0.88	0.66	1.17
2020	2013	1.18	0.90	1.56
Inspection number (>100 vs. <100)				
2016	2013	0.14	0.87	1.51
Data entry and record keeping time (>50% vs. <50%)				
2016	2013	0.88	0.59	1.31
2020	2013	1.0	0.68	1.48

^*^ *p* < 0.05.

^**^ *p* < 0.001.

^***^ *p* < 0.0001.

The Cochran–Armitage trend test found a significant decreasing trend in female biosafety professionals >11 years (*z* = −2.07, *p* < 0.05), while male biosafety professionals saw an increase over the same period.

Compared with respondents older than 39, the age ranges from 20 to 39 saw a significant decrease over the years (*Cochran–Armitage trend test z* = –3.48, *p* < 0.001). However, there is a significant increasing trend for respondents older than 39 over the same period. Logistic regression results indicate a gradual increase in the likelihood of biosafety professionals being over 39 years old over the past 11 years. Specifically, the odds of being >39 years old were 2.05 times higher for respondents in 2023 compared with respondents in 2013 (*OR* = 2.05, *p* < 0.001). In addition, respondents in 2024 had 1.76 times higher odds of being >39 years old compared with 2013 (*OR* = 1.76, *p* < 0.05).

The Cochran–Armitage trend test found a significant increasing trend in the biosafety professionals with >15 years of experience >11 years, whereas those with less years of experience saw a decrease. Logistic regression results indicate a gradual increase in the likelihood of biosafety professionals having >15 years of experience over the years. Specifically, compared to respondents in 2013, the odds of having >15 years of experience were 2.04 times higher for respondents in 2023 (*OR* = 2.04, *p* < 0.001). In addition, respondents in 2024 had 1.53 times higher odds of having >15 years of experience compared to those in 2013 (*OR* = 1.53, *p* < 0.05).

There is a significant increasing trend over the years (*z* = 5.77, *p* < 0.0001) for respondents with doctorate degree while respondents with no doctorate degree seeing a significant decrease trend over the same period. Logistic regression results indicate a gradual increase in the likelihood of biosafety professionals holding a doctorate degree over the years. Specifically, compared with respondents in 2013, those in 2023 had 2.5 times higher odds of having a doctorate degree (*OR* = 2.5, *p* < 0.0001). In addition, respondents in 2024 had even higher odds of holding a doctorate degree (*OR* = 2.77, *p* < 0.0001).

There is a significant decreasing trend over the years (*z* = −9.47, *p* < 0.0001) for respondents earning <$100,000 USD annually, while respondents earning >$100,000 USD annually saw a significant increase trend over the same period. Logistic regression results show that over the years, the odds ratios suggest a gradual increase in the likelihood of biosafety professionals earning >$100K compared with 2013. Specifically, compared with respondents in 2013, respondents in 2020 had significantly higher odds of earning >$100K (*OR* = 1.68, *p* < 0.001, and respondents in 2023 had significantly higher odds of earning >$100K (*OR* = 2.04, *p* < 0.001), and respondents in 2024 also had higher odds of earning >$100K (*OR* = 1.53, *p* < 0.05).

There is a significant increasing trend over the years (*z* = 3.57, *p* < 0.001) for respondents spending <50% of their time on biosafety responsibility while respondents spending >50% of their time on biosafety responsibility saw a significant decrease trend over the same period. Logistic regression results show that over the years, the odds ratios suggest a gradual increase in the likelihood of biosafety professionals’ respondents spending <50% of their time on biosafety responsibility compared with 2013. Specifically, compared with respondents in 2013, respondents in 2023 had significantly higher odds of spending <50% of their time on biosafety responsibility (*OR* = 1.53, *p* < 0.05).

There is a significant decreasing trend over the years (*z* = −4.94, *p* < 0.001) for respondents having ≤3 people in their biosafety team while respondents having >3 people in their team saw a significant increase trend over the same period. Logistic regression results show that over the years, the odds ratios suggest a gradual increase in the likelihood of biosafety professionals’ respondents having >3 people in their team compared with 2013. Specifically, compared with respondents in 2013, respondents in 2020 had significantly higher odds of having >3 people in their team (*OR* = 1.73 *p* < 0.0001), and respondents in 2024 had significantly higher odds of having >3 people in their team (*OR* = 1.95, *p* < 0.0001).

There is no trend observed in White and Other Race of biosafety professionals (*z* = 0.82, *p* = 0.41) and their ethnicity (*z* = 0.43, *p* = 0.67) >10 years. Similarly, there is no trend observed in biosafety professionals across different institution categories >10 years (*z* = −0.06, *p* = 0.95).

### Regression to Predict Income

Due to inconsistencies in survey questions across the 4 years studied, variables such as data entry responsibility, percentage of job biosafety responsibilities, and direct reports—previously identified as significant predictors of income—were excluded from the current regression model. Instead, the model focused on the sociodemographic variables of gender, age, education level, and years of experience and was rerun separately for each year on income and inflation adjusted income (Table 7).

**Table 7.** Regression model on income of sociodemographic variables

	Income				Adjusted income			
	2013	2016	2020	2024	2013	2016	2020	2024
B_1_	4844.57	11,539.00^*^	13127^***^	9064.04	6491.4	15,008^*^	15,832^***^	9064.04
B_2_	1450.2		7566.16^***^	6363.96	1943.15		9215.33^***^	6363.96
B_3_	10,183^***^	12,683.00^***^	10,305^***^	14,727^***^	13,645^***^	16495^***^	12,428^***^	14,727^***^
B_4_	10,885^***^	6614.54	13,899^***^	19,646^***^	14,586^***^	8602.64	16,763^***^	19,646^***^
*F*-value	20.2	14.84	46.32	14.42	20.2	14.84	46.32	14.42
R²	0.22	0.08	0.27	0.22	0.22	0.08	0.27	0.22
Adjusted R²	0.21	0.08	0.27	0.21	0.21	0.08	0.27	0.21

B_1_: Gender (male as 1 female as 0), B_2_: age (20–29 as 1 to 70 or more as 6), B_3_: years of experience (<1 year as 1 to >15 year as 5), B_4_: level of education (high school degree as 1 to doctorate as 4).

^*^ *p* < 0.05.

^**^ *p* < 0.001.

^***^ *p* < 0.0001.

The 2013 model explained 22% of the variance in income (*R^2^* = 0.22, *adjusted R^2^* = 0.21), producing a significant *F*-test (*F* = 20.2, *p* < 0.0001). The 2016 model, which excluded the age variable due to incompatible data, accounted for 8% of the variance (*R^2^* = 0.08, *adjusted R^2^* = 0.08) with a significant *F*-test (*F* = 14.84, *p* < 0.0001). The 2020 model explained 27% of the variance (*R^2^* = 0.27, *adjusted R^2^* = 0.27), again producing a significant *F*-test (*F* = 46.32, *p* < 0.0001). The 2024 model explained 22% of the variance (*R^2^* = 0.22, *adjusted R^2^* = 0.21), with a significant *F*-test (*F* = 14.42, *p* < 0.0001). The proportion of variance explained by these sociodemographic variables remained consistent in 2013, 2020, and 2024 but not in 2016, underscoring *the importance of age as a predictor of income*, particularly when age was excluded from the 2016 model.

Beta coefficients were analyzed to assess the impact of each variable annually, allowing for comparisons over time. Gender was a significant predictor of income in 2016 and 2020, with males expected to earn >$11,000 ($15,000 after inflation adjusted) more than females (*p* < 0.05). Age significantly predicted income only in 2020 (*p* < 0.05), where older biosafety professionals were projected to earn >$7500 ($9215 after inflation adjusted) more than their younger counterparts. However, age was not a significant predictor in 2013 or 2024. Experience consistently predicted income across all years (*p* < 0.0001), with professionals expected to earn approximately $10,183 ($13,645 after inflation adjusted) more in 2013 and $14,727 more in 2024 for every additional five years of experience. Education level was a significant predictor in 2013, 2020, and 2024 (*p* < 0.0001), with each higher degree associated with an income increase of ∼$10,000 (14,000 after inflation adjusted) in 2013, rising to nearly $20,000 in 2024.

While the individual year-specific regressions above focused on evaluating the influence of some sociodemographic factors including gender, age, years of experience, and level of education on income within 4 years (2013, 2016, 2020, and 2024), the linear regression including year as dependent variable allowed the assessment of temporal trends and the overall effect of year-to-year changes on income (Table 8).

**Table 8.** Regression model on income of sociodemographic variables

	Income	Adjusted income
	*B*	*B*
Gender	6565.69^*^	8066.77^*^
Age	3433.97^*^	4157.19^*^
Experience	8720.632^***^	10,664^***^
Education	10,142^***^	12,598^***^
Percentage of biosafety duty	−2148.61^*^	−2725.22^*^
Biosafety team	553.11	699.49
Reporting employees	5740.68^***^	7252.85^***^
Data entry and record keeping duty	−5773.13^***^	−7018.79^***^
Year	5296.04^***^	434.25
*F*-value	46.66^***^	43.41^***^
R-square	0.35	0.33
Adj R-Sq	0.34	0.33

^*^ *p* < 0.05.

^**^ *p* < 0.001.

^***^ *p* < 0.0001.

Two linear regression models were developed to examine the predictors of income. The first model used actual income for each year as the dependent variable, while the second model used inflation-adjusted income. The predictors included gender, age, years of experience, education level, percentage of time spent on biosafety responsibilities, the number of employees reporting to the respondents, the size of the biosafety team, the percentage of time spent on data entry and record keeping, and the year variable. The results indicated that, with the exception of the number of professionals in the team, all variables significantly predicted income in both models (*p* < 0.0001). However, in the second model, which adjusted for inflation, the year variable did not significantly predict income (*p* = 0.77), suggesting that year-to-year variations in income may be largely attributable to inflationary effects. In addition, the beta coefficients for most predictors were higher in the inflation-adjusted model, indicating that their impact on income is more pronounced when inflation is accounted for. Gender, age, years of experience, education level, and the number of employees reporting had a positive relationship with income (*B* > 0), while the percentage of time spent on biosafety responsibilities and data entry had a negative relationship with income (*B* < 0). The first model explained 35% of the variance (*R^2^* = 0.35, *adjusted R^2^* = 0.34) with a significant *F*-test (*F* = 44.79, *p* < 0.0001). The second model accounted for 33% of the variance (*R^2^* = 0.33, *adjusted R^2^* = 0.33) and also produced a significant *F*-test (*F* = 43.41, *p* < 0.0001).

## Discussion

This decade-long analysis of biosafety professionals reveals significant shifts in the workforce’s demographic and socioeconomic characteristics. Data for this analysis were obtained through surveys administered to biosafety professionals across various institutions over the years. However, potential biases may exist due to missing or inconsistent data and variability among survey respondents. Despite these limitations, the results highlight critical trends with far-reaching implications for the profession’s development and its capacity to meet emerging challenges in biosafety and biosecurity.

Our 10-year study of biosafety professionals’ age distribution reveals significant shifts within the field. In 2013, the workforce was dominated by individuals aged 50–59 years. By 2024, the 40–49 age group became the most prevalent, indicating an influx of middle-aged professionals over the past decade. This shift likely reflects the field’s growing appeal to experienced professionals seeking long-term careers. Another explanation for this could be due to the aging workforce, as the professionals who were aged 30–39 years in 2013 have advanced into their 40s. The retirement of older professionals in their 50s may also contribute to this shift. The professional aged 30–39 years in 2013 stayed in the field long enough to become the biggest workforce in 2024. Meanwhile, the proportion of professionals under 39 has decreased, suggesting challenges in attracting and retaining young talent in the field, possibly due to limited growth opportunities for less experienced individuals.^20,21^

**Figure 1.**
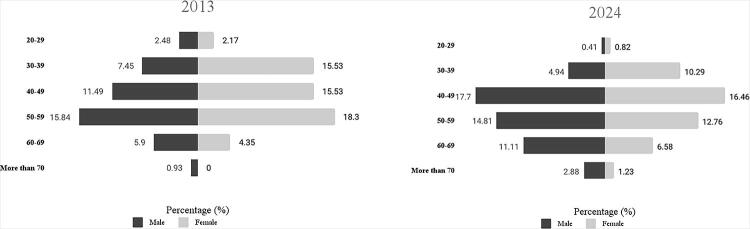
Age and gender distribution of biosafety professionals in 2013 and 2024.

A closer examination of the population pyramid (Figure 1) shows a notable increase in the proportion of men within the 40–49 age group, highlighting a strong influx of male professionals. Conversely, in the 50–59 age group, there is a significant decline in the number of women, while the number of men has only slightly decreased. This trend suggests that men are more likely to remain in the field as they age, whereas women tend to exit the workforce in their 50s and 60s.^22^

**Figure 2.**
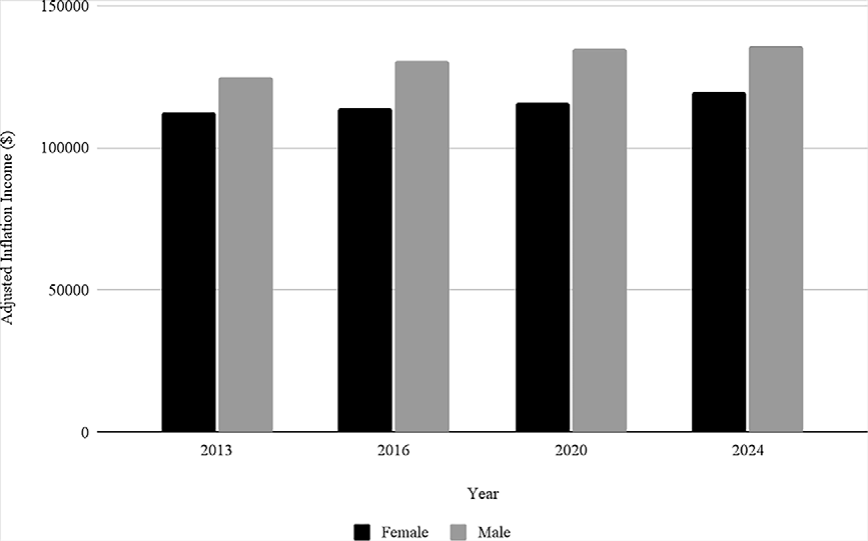
Difference in adjusted income of male and female biosafety professionals by year.

One possible explanation for the decreasing number of women in the biosafety field during their 50s and 60s emerges from the compensation data we collected (Figure 2 and Table 9). While there is no significant difference in income between men and women in the 20–49 age groups, a substantial income disparity becomes evident in the 50+ age group, where men earn, on average, >$20,000 more than women. This gap persists even when controlling for factors such as experience and education, with a significant income difference in the 50–59 age group despite similar qualifications. This disparity in compensation may contribute to the higher exit rate of women from the field, as unequal pay could lead to dissatisfaction and the decision to leave the workforce earlier than their male counterparts.^21^ In addition, this gender disparity may stem from challenges such as work-life balance, earlier retirement, and other factors disproportionately affecting women in the biosafety profession.^23^^,^^24^ Women often take on roles as sandwich caretakers for the overlapping child and elder caregiving responsibilities.^25^ According to a Wells Fargo report,^26^ 1.9 million women aged 55 years and older were out of the labor force due to family obligations—seven times the number of men in the same age group. In the biosafety profession, the odds ratio (male vs. female) comparing 2024 with 2016 is 1.38 and statistically significant, indicating a higher likelihood of men remaining in or entering the profession during this period. Among those aged 50–59 years, the percentage of women decreased from 18.32% in 2013 to 12.76% in 2024, while the percentage of men in this age group only slightly declined from 15.84% in 2013 to 14.81% in 2024. Both men and women experienced unprecedented employment declines during the pandemic, but the drop was steeper for women.^27^ Although employment rates for both genders recovered after the pandemic, the overall percentage change was low. Based on the study of older workers in the United Kingdom, it appears that the decision for many older women to retire or not return to the workforce was influenced by a variety of factors, including poor health, the natural conclusion of their working life, and the broader impact of COVID-19, such as changes to work demands, practices, and concerns about personal safety in the workplace.^28^

**Table 9.** Differences of income of biosafety professionals by gender in different groups

Group	Female	Male	*F*-value
Age 50–59	$121,519.11	$148,288.95	16.55^***^
Age 50–59 and doctorate degree	$134,576.54	$163,267.15	4.39^*^
Age 50–59 and master degree	$132,682.36	$152,129.22	3.86
Age 50–59 and >15 years	$133,930.40	$158,053.91	7.22^*^
Age 50–59 and 5–10 years	$102,388.88	$127,467.93	2.69
Age 50–59 and 10–15 years	$117,192.68	$124,382.00	0.52
Age 60–69 and doctorate degree	$159,660.29	$163,943.61	0.06
Age >70 and doctorate degree	$222,000.00	$163,471.25	0.99

^*^ *p* < 0.05.

^**^ *p* < 0.001.

^***^ *p* < 0.0001.

**Figure 3.**
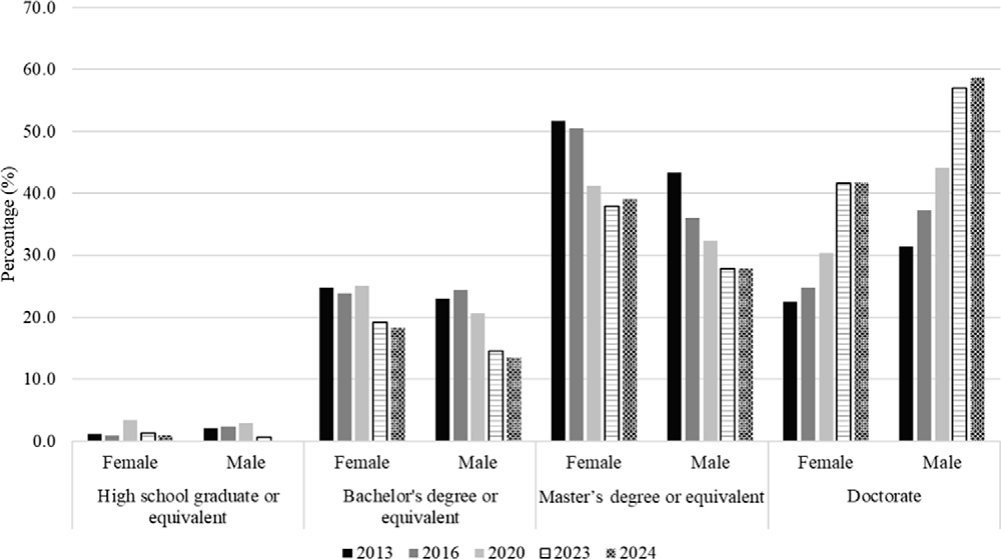
Percentage of biosafety professionals by different educational levels by gender from 2013 to 2024.

The shift in educational attainment among biosafety professionals is another critical finding. The proportion of individuals with doctoral degrees has significantly increased, reflecting the growing demand for advanced expertise in the field (Figure 3). However, the increases in the doctorate degree is greater among men than for women. This trend highlights the importance of higher education in biosafety, where the complexity of responsibilities and the need for specialized knowledge are paramount. However, the decreasing proportion of professionals with only a bachelor’s or master’s degree may suggest a narrowing pathway into the profession, potentially excluding individuals who could contribute valuable perspectives and skills.

The rising proportion of biosafety professionals holding doctoral degrees and having >15 years of experience reflects a workforce that is becoming increasingly specialized and experienced. However, with years of experience and age being moderately correlated (*corr* = 0.5) among surveyed professionals, concerns arise regarding the health, safety, and workload management of an aging workforce.^29^ This trend also underscores the challenge of sustaining the field amid declining numbers of younger professionals entering the profession. To address this, there is an urgent need for educational strategies that integrate biosafety-focused programs into academic curricula, equipping young professionals with practical knowledge. In addition, recruitment strategies, complemented by onsite training, are essential to ensure the continued infusion of new talent and the advancement of the profession.^30^

The analysis reveals significant racial disparities within the biosafety profession, where the workforce remains predominantly White with minimal representation from other racial and ethnic groups over a decade. This underrepresentation of racial and ethnic minorities in the biosafety field reflects broader trends seen in various healthcare professions, where lower educational attainment, limited access to educational pathways, financial support, and role models contribute to these disparities.^31–33^ The lack of significant change in this area over the past decade indicates that efforts to diversify the workforce have not been sufficiently effective, underscoring the need for targeted initiatives to enhance diversity, equity, and inclusion within the profession.^34^^,^^35^

**Figure 4.**
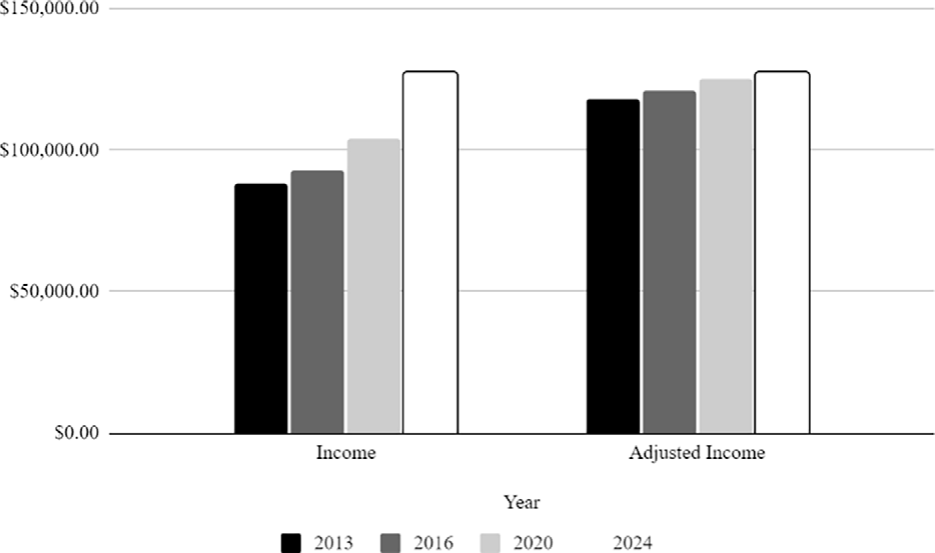
Average actual income and inflation-adjusted income of biosafety professionals from 2013 to 2024.

Income trends among biosafety professionals reveal an upward trajectory, particularly in the proportion of individuals earning >$100,000 annually. While this rise in nominal income may indicate an increasing recognition of the value biosafety professionals bring to their roles, the analysis also shows that, when adjusted for inflation, the real income growth has been modest and statistically insignificant (Figure 4). Coupled with other trends, such as rising levels of education, experience, and role complexity, the data suggest that biosafety professionals are taking on greater responsibilities and becoming more specialized and experienced yet are not receiving higher compensation beyond inflation adjustments. This could have implications for job satisfaction and retention.^36^^,^^37^

The regression analyses further illuminate the predictors of income in the biosafety profession. Across the years, gender, age, education level, and years of experience have consistently influenced earnings. Gender disparities in income were particularly notable in 2016 and 2020, where males were found to earn significantly more than their female counterparts. This finding underscores the need for ongoing efforts to address gender-based income inequality in the profession.^38^ Age and experience emerged as significant predictors of income,^14–17^ with older and more experienced professionals earning higher salaries, likely due to their accumulated knowledge and seniority. The increasing importance of educational attainment, particularly the possession of a doctoral degree, as a predictor of income in recent years, suggests that advanced education is becoming more critical for career advancement in biosafety.

**Figure 5.**
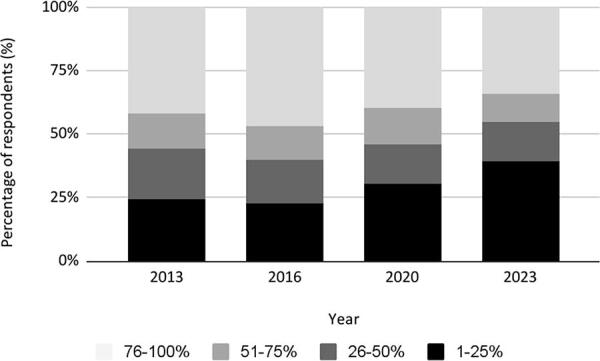
Distribution of percentage of time spent on biosafety responsibility of biosafety professionals from 2013 to 2023.

**Figure 6.**
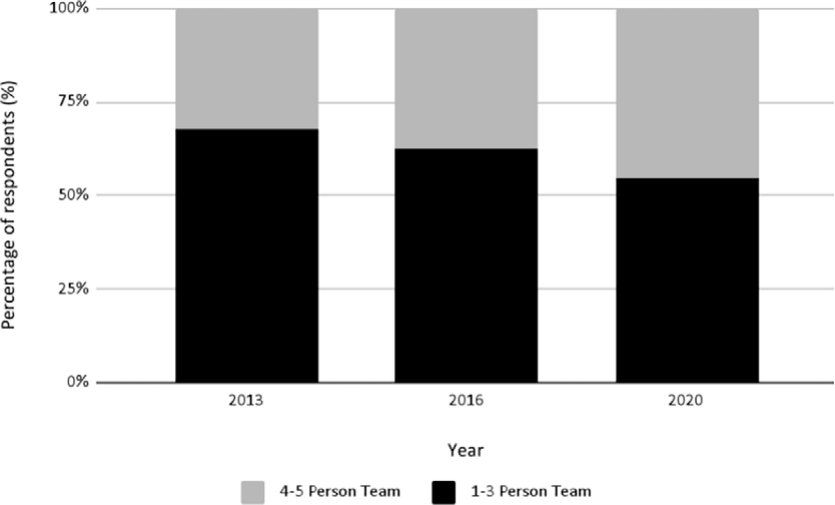
Distribution of the size of biosafety team from 2013 to 2020.

In addition to these demographic and socioeconomic factors, the analysis also reveals changes in the workplace and job characteristics of biosafety professionals. There is a noticeable trend toward larger biosafety teams and a broader distribution of biosafety responsibilities across different roles within organizations (Figures 5 and 6). This shift may reflect the growing complexity of biosafety operations and the increasing integration of biosafety responsibilities with other functions, such as biosecurity and supervisory roles. The likelihood of having larger biosafety teams within institutions was higher in 2020 and 2024 compared with 2013 and 2016. However, the likelihood of professionals dedicating their time solely to biosafety tasks decreased in 2023 compared with 2013 and 2016. The COVID-19 pandemic has likely accelerated these changes, as the demand for biosafety expertise has increased in response to the global health crisis as well as the rapid growth of the bioeconomy worldwide.^39–42^

The demographic and socioeconomic trends identified in this study highlight both the progress and challenges facing the biosafety profession. The aging workforce, increasing educational attainment, and rising income levels reflect a profession that is becoming more specialized and valued. However, the declining representation of younger professionals, ongoing gender and racial disparities, and modest real income growth indicate areas where targeted interventions are needed. To ensure the sustainability and diversity of the biosafety workforce, stakeholders must prioritize policies and programs that attract and support a broader range of professionals, particularly younger individuals and those from underrepresented groups. By addressing these challenges, the biosafety profession can better meet the demands of an increasingly complex and dynamic field, ensuring its continued contribution to public health and safety.

### Limitation and Future Research

This study acknowledges potential biases in survey responses, particularly the likelihood that professionals who had previously participated in the ABSA surveys were more inclined to continue participating due to their recognition of the survey’s importance. This could have led to an overrepresentation of experienced biosafety professionals, while younger professionals, who may have been less aware of the survey, were underrepresented. In addition, the inconsistency in survey questions and answers across different years may have contributed to missing data, limiting the ability to make direct comparisons over time.

Future research should prioritize increasing awareness and participation among younger biosafety professionals to ensure a more representative sample of the workforce. Efforts should be made to engage these professionals early in their careers. In addition, developing a standardized survey questionnaire that consistently captures key demographic, socioeconomic factors, and job responsibilities across different years would enhance the reliability and comparability of longitudinal studies. This standardization would reduce the risk of missing data and improve the validity of trend analyses.

Given the significant changes observed in the biosafety workforce between 2013–2016 and 2020–2024, further investigation into the potential impact of COVID-19 pandemic on biosafety professionals is warranted. Understanding how COVID-19 affected biosafety job responsibilities, stress levels, job satisfaction, and career development within this field could provide valuable insights for future workforce planning as well as for preparation for future emerging global health crises.

The changes in demographics and shifts in biosafety professionals identified in this study can serve as a foundation for further research into long-term career progression and mobility within the profession. Future studies could explore how this profession’s responsibilities evolve, the availability and impact of training, and career advancement opportunities, while also examining how these aspects vary across different demographic groups.

Finally, while factors in the studied regression model such as age, education, experience, gender, and job responsibilities were identified as predictors of salary, they accounted for only 34% of the variance in the regression model. This suggests that other factors, potentially unmeasured in this study, contribute significantly to salary determination. Future studies should consider including additional variables such as institution type, geographic work location, professional credentials, and organizational structure to develop a more comprehensive predictive model of salary within the biosafety profession.

## Conclusion

This study highlights significant changes in the biosafety field over the past decade (2013–2024), reflecting shifts in both workforce demographics and professional characteristics. The proportion of older and more experienced biosafety professionals has steadily increased as has the percentage of individuals holding doctoral degrees. However, the proportion of women in the workforce has declined, and White, non-Hispanic individuals continue to dominate the field with limited changes in racial and ethnic diversity. In addition, there has been a decrease in the number of professionals dedicating their time solely to biosafety responsibilities, accompanied by an increase in biosafety team sizes. While income has risen significantly over time, the increase is less pronounced when adjusted for inflation. Education level and experience remain strong predictors of income, whereas the influence of age and gender on salary has fluctuated.

Biosafety is a promising field that attracts many experienced middle-aged professionals seeking long-term careers. However, the decline in the number of professionals under 39 poses a potential risk of labor shortages in the future. This trend underscores the importance of implementing targeted recruitment and training strategies to attract younger individuals to the field. Integrating biosafety-focused educational programs into academic curricula can equip future professionals with the practical knowledge needed to enter and thrive in this field. Every workplace should also provide opportunities for young professionals to grow in the field by offering targeted training, promoting career advancement opportunities, and fostering mentorship programs. Organizations and institutions should prioritize diversity initiatives and foster inclusive environments to attract and retain professionals from diverse backgrounds.

Understanding the impact of demographic factors on income over time offers valuable insights for professionals making career decisions and for recruiters monitoring trends and growth within the biosafety profession. By examining trends in the sociodemographics of biosafety professionals and changes in workforce responsibilities, institutions and policymakers can develop responsive strategies to adjust policies and recruitment processes, fostering a more balanced and diverse workforce capable of addressing the complex challenges of modern biosafety work.

## Data Availability

The data that support the findings of this study are available from the corresponding author upon reasonable request. Restrictions may apply to the availability of the data due to privacy or ethical considerations.
